# Muography for a dense tide monitoring network

**DOI:** 10.1038/s41598-022-10373-y

**Published:** 2022-04-25

**Authors:** Hiroyuki K. M. Tanaka

**Affiliations:** University of Tokyo, Tokyo, Japan

**Keywords:** Ocean sciences, Physical oceanography, Natural hazards, Experimental particle physics

## Abstract

Sub-hourly to seasonal and interannual oceanographic phenomena can be better understood with high special resolution and high frequency tidal observations. However, while current tidal measurements can provide sufficiently high observational density in terms of time, the observational density in terms of space is low mainly due to the high expense of constructing tide gauge stations. In this work, we designed a novel tide monitoring technique with muography that could be operated in near-shore basements (or similar structures on land below sea level) and found that more practical, stable, robust and cost-effective high-spatiotemporal-density tide measurements are possible. Although the time resolution, sensitivity, and the distance between the detectors and the shorelines are tradeoffs, hourly and annual sensitivity (ability to detect the tide height variations) of less than 10 cm and 1 mm can be statistically attained, respectively. It is anticipated that the current muographic technique could be applied as an alternative, cost-effective and convenient dense tidal monitor network strategy in coastal areas worldwide.

## Introduction

**Figure 1:**
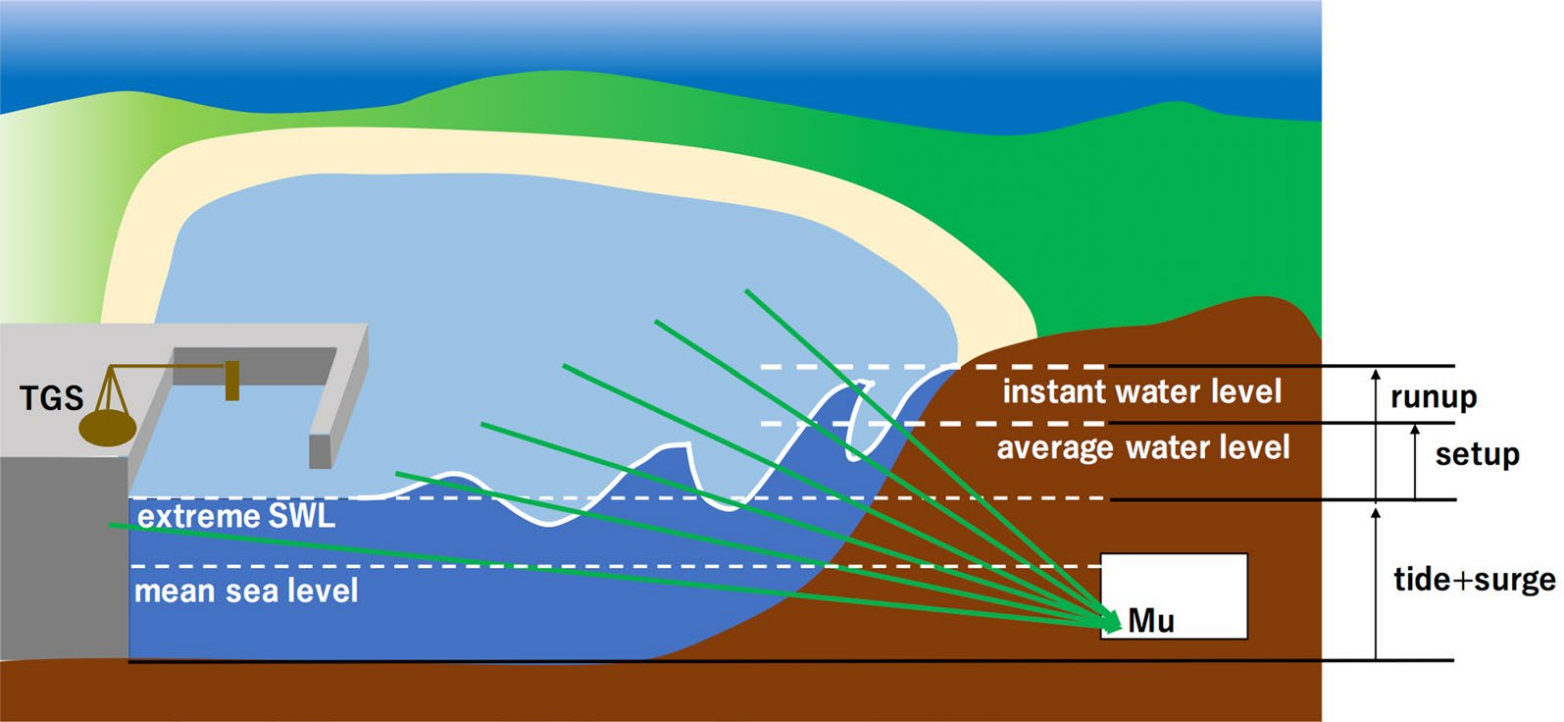
Conceptual view of muography for tide monitoring. The label TGS stands for a tide gauge station, and Mu indicates the location of a muograph (muographic observation and recording device). Green arrows indicate muon trajectories. HKMT drew this image with Microsoft PowerPoint software and holds the copyright.

Recently flood hazards have increased, which have been exacerbated by a rising global mean sea level due to land ice melting and ocean warming^1–3^. One of the most important factors to quantify the extent of this increase is the flood hazard magnification rate^4^. Utilizing data from several years of tide gauge observations of various extreme weather and flooding events has been the most common way to determine this amplification^5–7^. Tidal levels have been measured and monitored in order to obtain reliable sea-level information including tides, surges, waves, and relative sea-level rise. Such information is essential for coastal communities since coastal flooding is increasingly occurring in many areas^8,9^. Also, understanding of the regional tide streams in the inner bay is important for the safety of navigation and environmental assessments, as well as to improve assessments of regional seawater circulation types and pollution distribution, and tidal flow fields studies, which have been numerically modeled in various regions^10–14^. In order to create accurate modeling for both forecasting storm surges and estimating tidal fields spatiotemporally, high density tide level information is required as a boundary condition. However, since tide gauge stations (TGSs) are expensive and usually have to be deployed in wave-sheltered harbors, these are only sparsely distributed even within large metropolitan bay areas^15^. For this reason, the tide level data have been interpolated to reproduce the continuity and smoothness of the tide level distribution^16^. Moreover, TGSs measure only Still Water Levels (SWLs) on the spot, and as a consequence, we tend to underestimate or exclude waves that include wave setup and wave run-up^17–19^(Fig. 1). These tide gauge-based estimates of the amplification of extreme water levels therefore are not always accurately assessing actual shoreline conditions outside the wave shelter. Caires et al.^20^ analyzed and extrapolated a long-term time series of still water level data measured by the Dutch Ministry of Transport, to find that extreme SWLs with heights of 354 cm, 411 cm, and 466 cm respectively occur at 100, 1000 and 10,000 year intervals. Marsooli and Lin^21^ investigated interactions between storm tides, surges, and waves for historical tropical cyclones (1988–2015) in the western North Atlantic Ocean, and found that the maximum wave setup was relatively large (tens of cm) in most coastal regions, but it did not always coincide with the peak storm tide. Abdalazeez analyzed the dataset provided by the European Centre for Medium-Range Weather Forecasts (ECMWF), and estimated that runups higher than 1.5 m are generated by wind waves with heights between 4.0 to 6.0 m^22^. Seenipandi et al.^23^ mentioned that the beach profile shape of coastal zones are highly suspectable to be changed with wave run-ups greater than 6.0 m.

Satellite-based radar altimetry provides a more global solution for analyzing tidal changes in wider areas. However, space–time resolutions depend on the satellite orbit and in particular, on its repeat period. The intertrack distance at the equator depends on the mission repeat cycle. Some examples include the following: an intertrack distance of 315 km for 10 days for Topex/Poseidon and Jason-1/2/3 missions, an intertrack distance of 104 km for 27 days for the Sentinel-3A/3B missions with one satellite and 53 km with Sentinel-3A and -3B working in tandem, and an intertrack distance of 80 km for 35 days for ERS-1/2, Envisat, and SARAL/AltiKa^24^. Also, the validity of measurements close to the coast is limited and may not accurately represent the coastal processes^25^. Global-navigation-satellite-system (GNSS) buoys using GNSS satellite positioning technology may solve this problem with faster time resolutions (30 s–1 day)^26^. However, installing buoys in the high maritime traffic areas is not practical. Moreover, the deployment and data transfer costs tend to be high. Ocean bottom sensors such as pressure and ultrasonic gauges provide tidal information in realtime^27^. However, since these sensors have to be directly located on the seafloor, preparation of infrastructures for electricity and data transfer are expensive. Moreover, pressure gauges have an intrinsic drift error, and the propagation time of the ultrasonic signals depend on solar radiation, seasonal cycles, mixing of the water due to sea currents, and the presence of rivers or waste waters^28^.

Recently, muography, which has been conducted from an underwater tunnel showed the potential to offer a practical real-time tide monitor without intrinsic drift errors, and without the requirement to provide infrastructures for electricity and data transfer^15^. Muons are produced during the interaction between primary cosmic rays and the nuclei in the Earth’s atmosphere. These muons are called cosmic-ray muons. On Earth, the muon flux reaches its maximum (~ 200 m^−2^ s^−1^sr^−1^) at an atmospheric depth of 300 gcm^−2^, and then slowly decreases as the muons pass through the additional atmospheric depth. The muon flux is ~ 90 m^−2^ s^−1^sr^−1^ at sea level^29^. The distance muons can traverse in materials is a function of the incident muon energy and average density along the muon path. This can be inferred based on known data of the topography and the open-sky muon spectrum. Once both the muon path length and the average density along the path are known, the densimetric thickness (*x*)^30^ can be calculated by multiplying them, and thus the minimum energy (*E*_c_) of muons that can penetrate through a material with this thickness can be determined. By integrating the open-sky spectrum from *E*_c_ to infinity, we obtain the expected flux of muons after passing through the target object. Inversely, if we place a detector underneath the target of interest and measure the muon flux, we can map out the densimetric thickness distribution as a function of azimuth and zenith angles^31^. Due to the penetrative and ubiquitous nature of cosmic-ray muons, they have been utilized as probes for muography and have been widely applied to visualizing the internal structure of gigantic objects. Muographs (muographic observation and recording devices) have been deployed to observe targets such as volcanoes^32–41^, rock overburdens^42–44^, and cultural heritage sites^45–48^ (typically being positioned below the targeted region) to record muographic images of these land objects, but as mentioned above more recently, the Hyper Kilometric Submarine Deep Detector (HKMSDD) realized the goal of underwater muography imaging at reasonable costs by installing muographs inside an underwater tunnel. However, underwater tunnels are not always available in coastal areas that would benefit the most from muography monitoring. In this work, an alternative to using underwater tunnels is proposed: HKMSDD muon sensor modules (HKMSDD-MSMs) could be deployed in coastal regions globally within available near-shore basements (or similar structures on land sufficiently below sea level), and could be applied as a convenient, dense and standalone tidal monitoring network or in tandam with another network.

## Results

### Principle

Galactic cosmic rays (GCRs) are accelerated by high energy events in our galaxy and before they arrive at Earth, they are deflected multiple times during their propagation, and lose their initial directional information. Muons are produced in the Earth's atmosphere via the collision between these GCRs and the Earth’s atmospheric nuclei. Due to different atmospheric thicknesses and density gradients for different GCR's arrival angles, the muon energy spectrum varies according to the different zenith angles. As a consequence, the vertical muon flux is higher than the horizontal flux, but the average energy of vertical muons is lower than the horizontal ones. The HKMSDD-MSM tide monitor utilizes these near horizontal muons.

**Figure 2:**
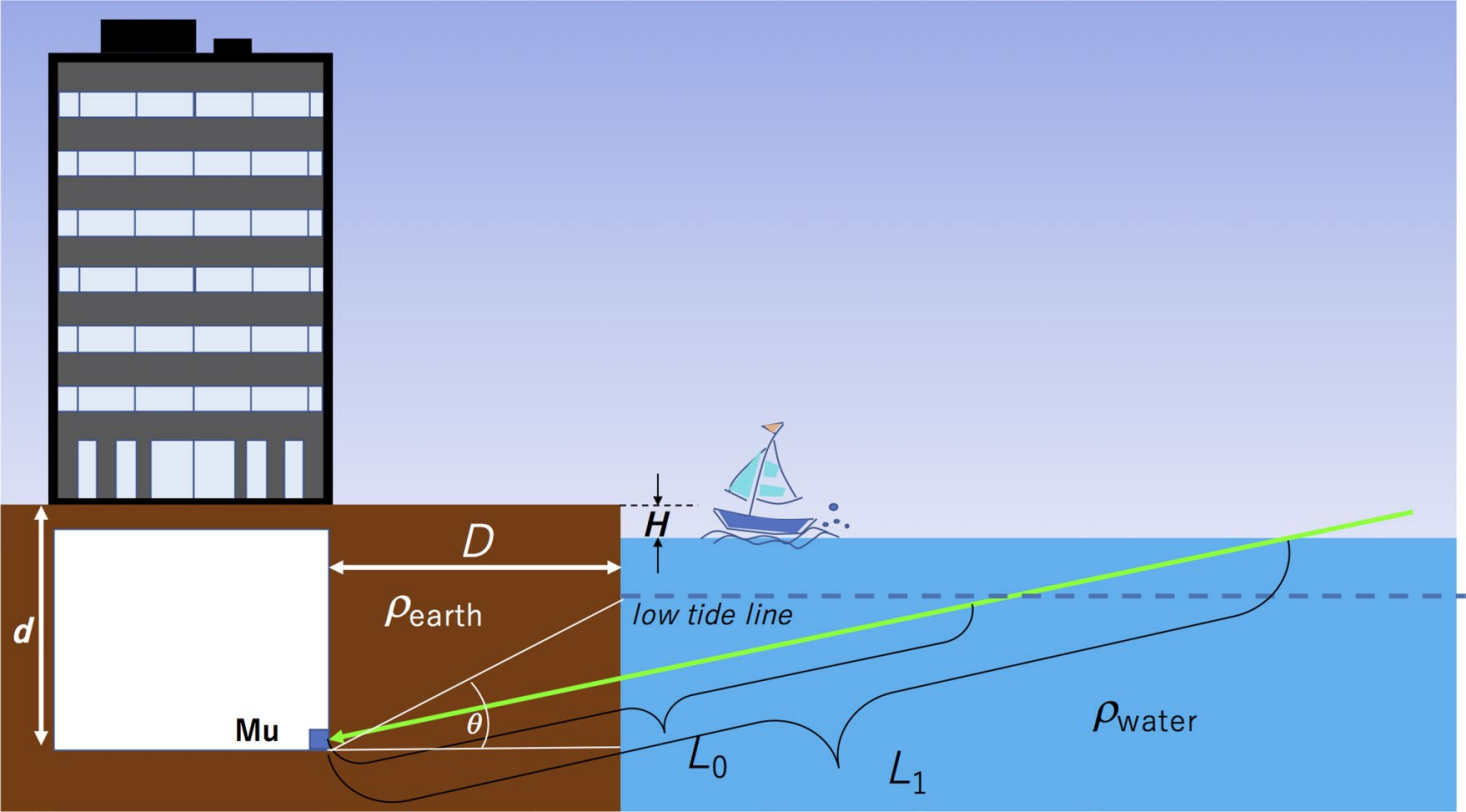
Principle of HKMSDD-MSM tide monitoring. The green arrow and the blue square labeled Mu indicate respectively the muon trajectory and the location of the HKMSDD-MSM. *L*_0_ and *L*_1_ respectively indicate 1. the muon's path length when the lowest waterline reached during the spring tide and 2. the muon's path length when sea levels rise due to surges, tsunamis, etc. HKMT drew this image with Microsoft PowerPoint software and holds the copyright.

Figure 2 shows the principle of HKMSDD-MSM tide monitoring. In this scheme, HKMSDD-MSMs are placed at near-shore locations below sea level such as basements of commercial buildings, subway stations, underground parking lots etc. As shown in Fig. 2, near horizontal muons would pass through seawater and land soil before arriving at the MSM. The total thickness of the materials that muons will traverse through before arriving at the MSM is:

1 $$L = D/\cos \theta \, + \left( {d - H} \right)/\sin \theta \;\left( {\text{m}} \right)$$

where *D* (m) is the distance between the MSM and the shoreline, *H* (m) is the land altitude measured from the lowest tide level, *d* (m) is the depth of the MSMs measured from ground level, and *θ* is the elevation angle. Here the average densities of the land soil (*ρ*_earth_) and seawater (*ρ*_water_) were respectively assumed to be 2.0 g cm^−3^ and 1.0 g cm^−3^. Since the tide level variations ∆*h* (m) only changes the second term of Eq. (1), as *D* increases, the MSM's sensitivity to the tide variations is degraded.

The muon flux observed at the MSM (*N*) can be calculated as follows. Once *L* is determined, the minimum muon energy (*E*_c_) that arrives at the MSM can be derived from the muon's energy range relationship in H_2_O and SiO_2_^49^. By integrating the open-sky muon energy spectrum^50–52^ over the energy range between *E*_c_ and infinity, we obtain the angular dependent integrated muon flux *I* (*θ*), where *θ* is elevation angle. By integrating *I* (*θ*) over the angular range between 0 and *Θ*, *N* is derived, where *Θ* holds the following relationship:

2 $$\tan \Theta = \left( {d - H} \right)/D$$

**Figure 3:**
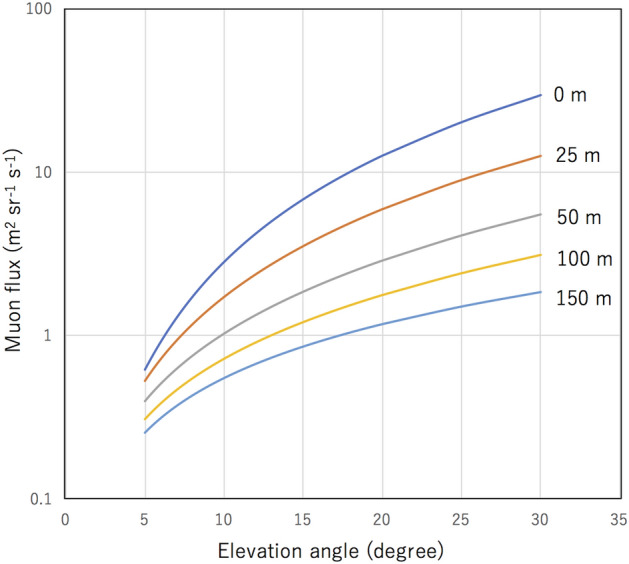
Integrated muon flux as a function of the elevation angle for different distances: 0 m (blue), 25 m (red), 50 m (gray), 100 m (yellow), and 150 m (light blue) between the MSM and the shoreline.

Figure 3 shows *I* as a function of the elevation angle (*θ* < *Θ*) for different *D*. As long as *θ* < *Θ*, the soil portion in *L* relies only on *D* and *θ*. Therefore, as the distance between the MSM and the shoreline increases, the number of muons that arrives at the MSM will decrease. As a consequence, the time resolution of the HKMSDD-MSM tide monitor will be degraded as the length of *D* increases.

### Case studies in Tokyo Bay

Urban underground spaces (UUSs) have various functions: storage, industry, transport, utilities and communications, and public use. In Tokyo, most of the underground facilities in the city areas are for public use. Tokyo uses more than 50% of UUSs for transportation including subways, highway tunnels, and stations, and almost 40% of UUSs for public spaces, shopping areas, parking lots, storages and industrial use^53^. Throughout its historical development, UUSs in Tokyo have progressed from shallow to deep soil layers. Therefore, inside UUSs, the supply of stable utility (electricity, gas and water) is one of the most important factors. In Japan, the UUSs for public use are equipped with a three-step power failure prevention system. In particular, there is a regulation that a UUS with a floor area exceeding 1000 m^2^ must be equipped with an independent emergency power generator by a UUS managing body. If the emergency power generator is shut down for some reason, it will be immediately replaced with a battery-operated system. Such a robust pre-installed infrastructure particularly designed for UUSs also offers an ideal space for stable and safe operations of muographic tide monitors even under extreme conditions such as severe storms and earthquakes. In order to install the HKMSDD-MSM tide monitor to a UUS, we need (A) a commercial electricity supply, (B) a network environment, and (C) one or more rooms with dimensions of at least a few m^2^. In most cities, a commercial electricity supply is available in UUSs, and a network infrastructure is also well organized in UUSs. For example, in the case of UUSs in Tokyo, it is relatively easy in this kind of location to arrange a new contract with a carrier to add one more internet line inside the building without additional costs. Regarding space requirements, if a large room (e.g., 100 m^2^) is available, installation of one HKMSDD-MSM would be sufficient, but if such a large room is not available, the size of each MSM would have to be smaller; hence in this case the number of smaller MSMs could be increased to attain a suitable detector area to collect the sufficient amount of muons, and consequently the device costs per station will be increased.

**Figure 4:**
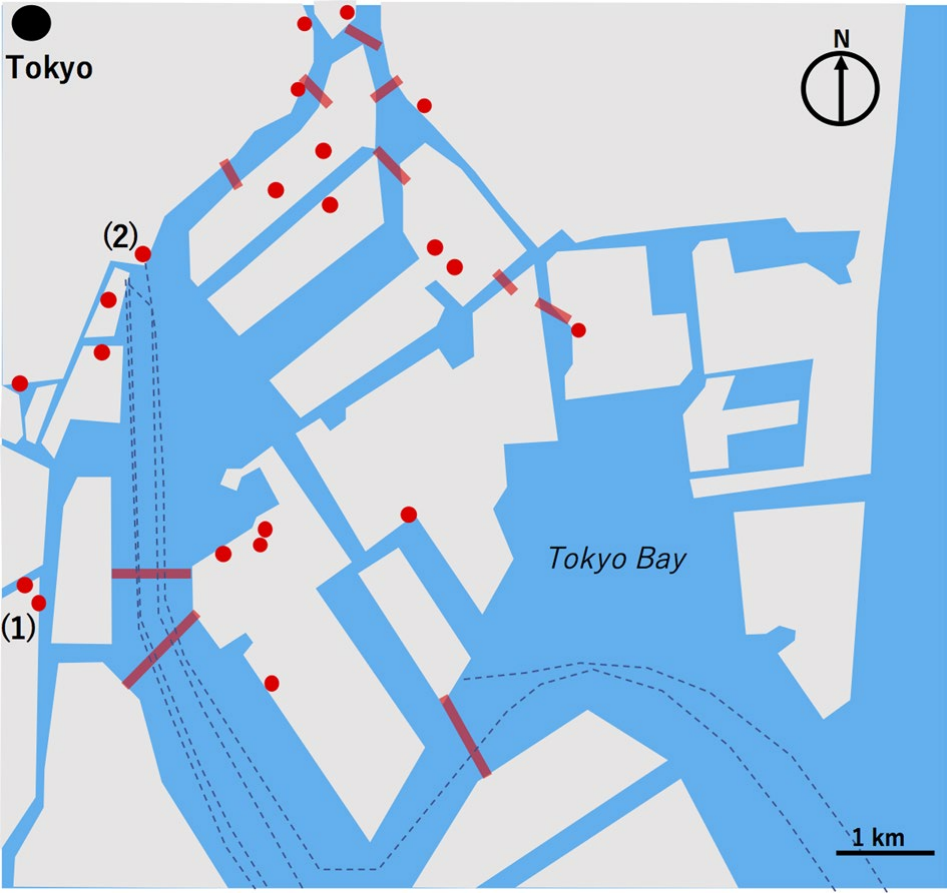
South-central Tokyo map showing the distribution of the Tokyo deep and large-scale urban underground spaces (UUSs) located in the regions within 200 m from the shorelines. Commercial UUSs including subway stations, shopping areas and parking lots (filled red circles) and underwater tunnels (red bold lines) are shown. Dashed lines indicate the ocean lines. The points (1) and (2) indicate the locations of the UUSs mentioned in the current modeling work. HKMT drew this map with Microsoft PowerPoint software and holds the copyright.

The south-central Tokyo map in Fig. 4 shows the distribution of Tokyo deep and large-scale UUSs (DLUUSs) located in the regions within 200 m from the shorelines. Here, the DLUUSs are defined as those having basement floors located below sea level with a floor size exceeding 1000 m^2^. The south-central part of Tokyo consists of the main land and more than 15 islands in the north part of Tokyo Bay. Most of these islands are connected by bridges, but lines on the ocean in Fig. 4 represent railway/motor underwater tunnels which connect these islands. A number of commercial skyscrapers were built on these islands, and some of them have UUSs reaching depths greater than 10 m from the ground surface. Since the elevation of these islands ranges between 1 and 7 m, these floors are located below sea level.

### HKMSDD-MSM

**Figure 5:**
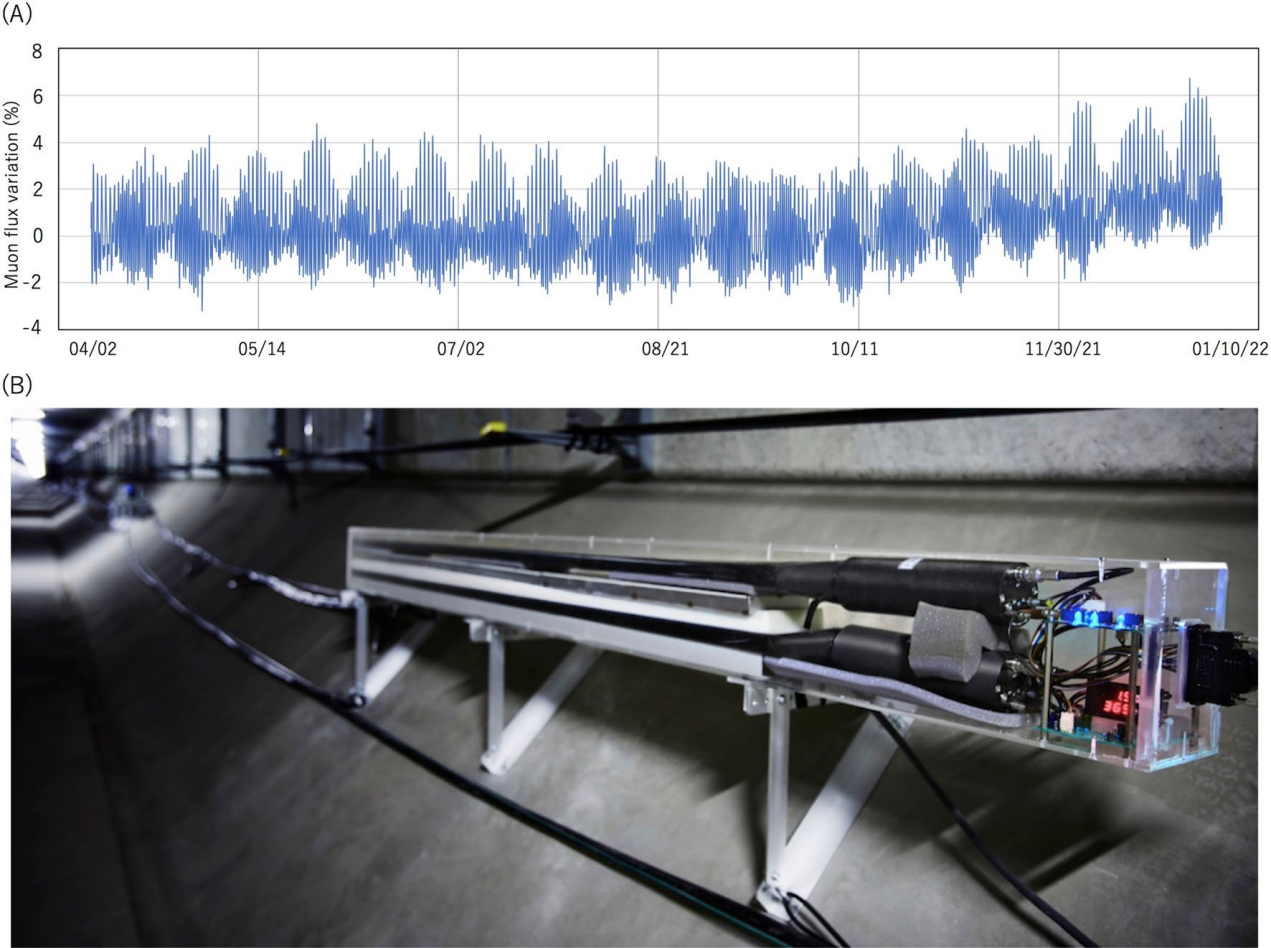
Tide variations recorded at TS-HKMSDD without any intermittency or measurement drift (**A**). A photograph of TS-HKMSDD MSM designed for collecting vertical muons is also shown (**B**). HKMT holds the copyright of the photograph.

The currently proposed muographic tide monitor is based on the successful, stable and maintenance free long-term observation conducted by an array of MSMs from the Tokyo-bay Seafloor HKMSDD (TS-HKMSDD) at one of the UUSs called Aquatunnel (underwater highway tunnel) in Tokyo bay^15^. As shown in Fig. 5A, since we started the stable operation mode of TS-HKMSDD in April, 2021, TS-HKMSDD has successfully recorded the tide level variations without any interruptions, and has continued to record the tide level variations without any intermittency or measurement drift. On the other hand, TGS observations frequently give erroneous data^54^. For instance, during TGS measurements, data can become suddenly corrupted with noise, or (due to mechanical problems) the moving parts of the gauge may lock up or malfunction^54^. Unlike tide gauges or buoys, HKMSDD doesn't have to be exposed to harsh environments and there aren't any mechanically moving parts in HKMSDD (Fig. 5B). This would also be the case for the proposed HKMSDD-MSMs setup; additionally, since UUSs such as commercial buildings or highway tunnels are already equipped with a robust electric and internet environment and each have a private power generation backup system, it is anticipated that muographic tide monitors would have the highest level of stability compared with other legacy tide level monitors.

**Figure 6:**
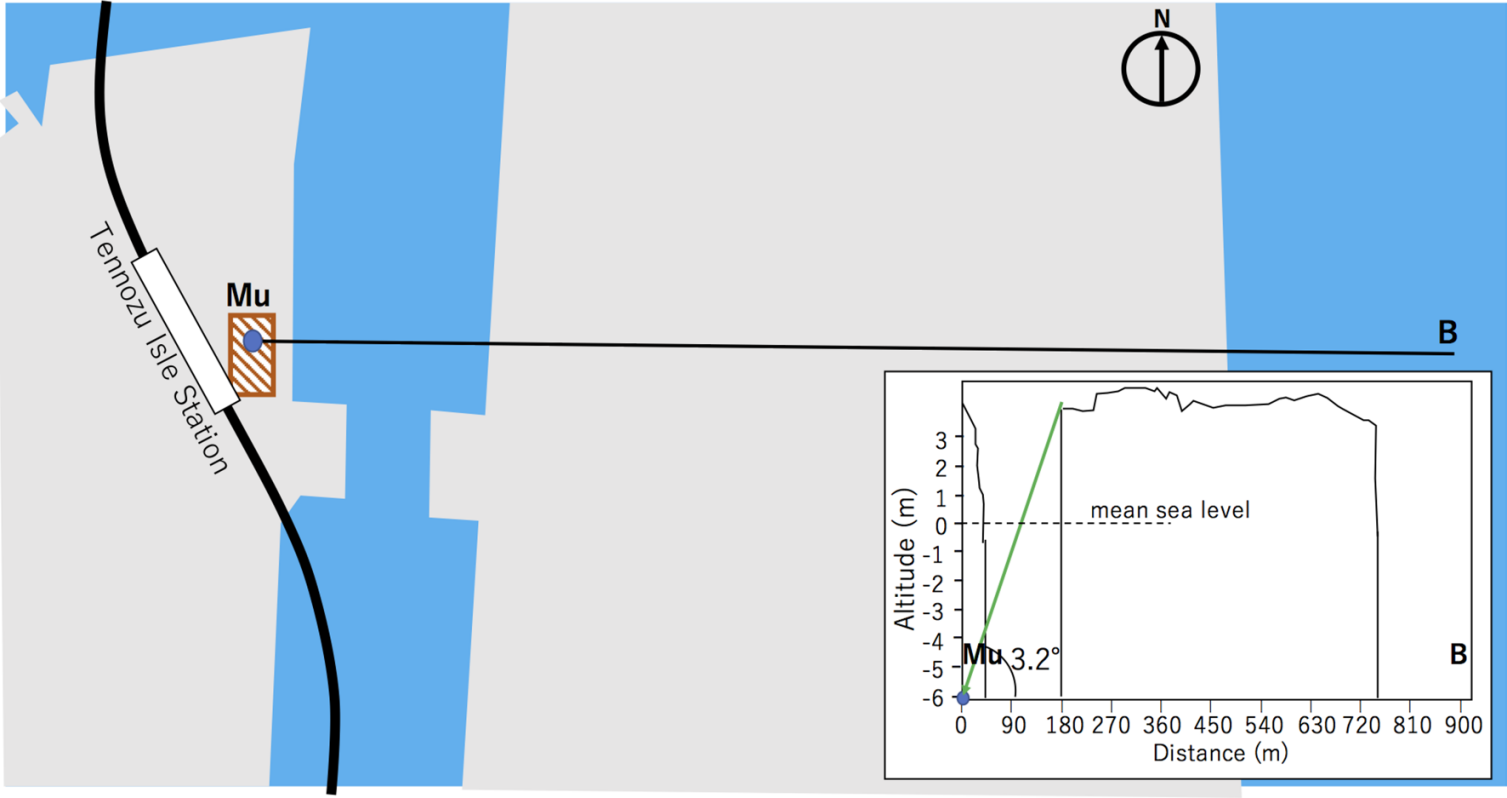
Enlarged map near the region of (1) in Fig. 4. The red shaded rectangle indicates a commercial building having a basement floor located 10 m below the ground surface. The label Mu indicates the location of the HKMSDD-MSM. The inset shows the cross-sectional view along the Mu-B line as an example of the vertical cross-sectional configuration of the measurement. The elevation scale is magnified by 50 times compared to the horizontal scale. HKMT drew this map with Microsoft PowerPoint software and holds the copyright.

**Figure 7:**
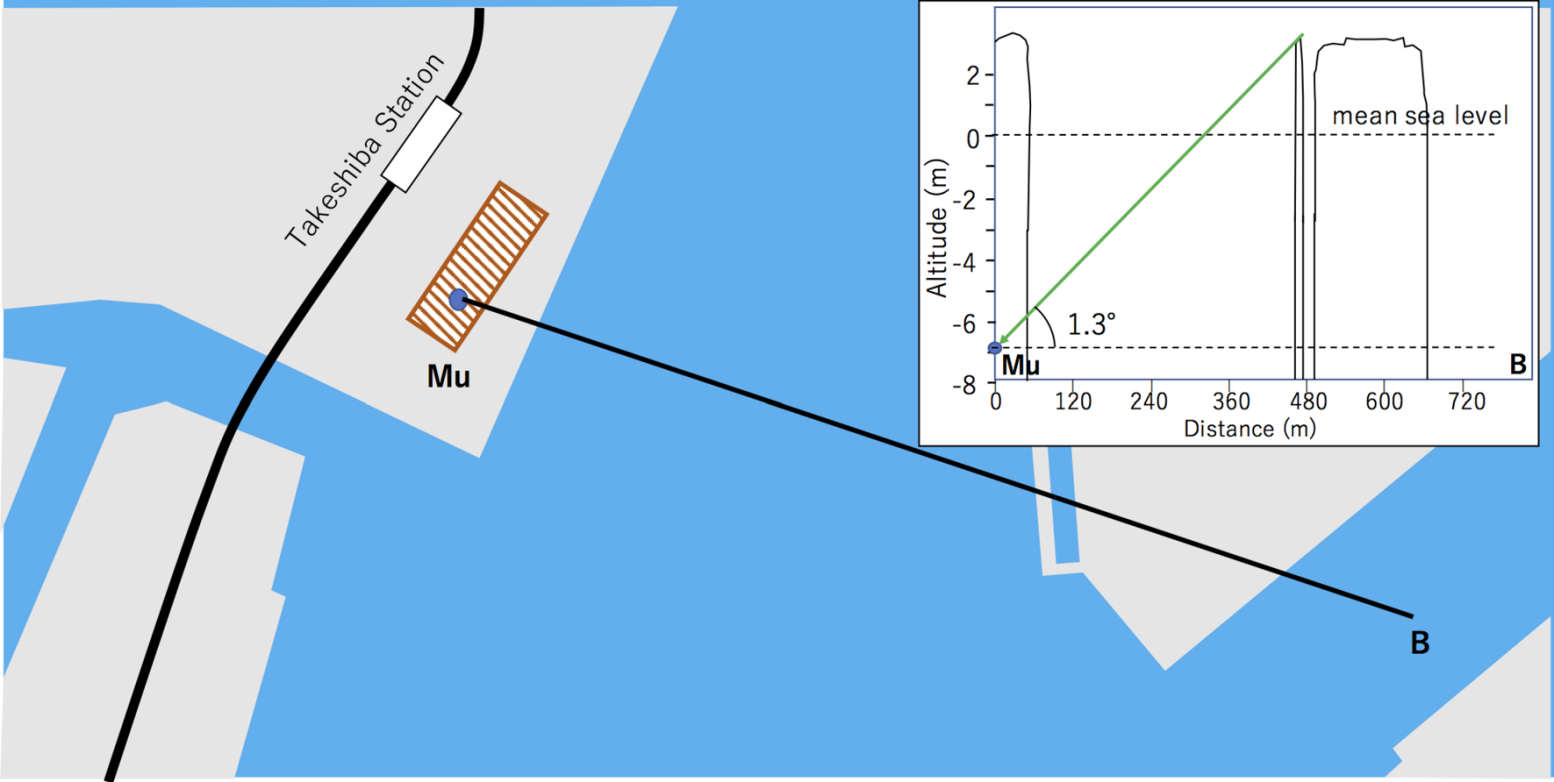
Enlarged map near the region of (2) in Fig. 4. The red shaded rectangle indicates a commercial building having a basement floor located 10 m below the ground surface. The label Mu indicates the location of the HKMSDD-MSM. The inset shows the cross-sectional view along the Mu-B line as an example of the vertical cross-sectional configuration of the measurement. The elevation scale is magnified by 50 times compared to the horizontal scale. HKMT drew this map with Microsoft PowerPoint software and holds the copyright.

Figures 6 and 7 show close and vertical cross-sectional views of some representative UUSs indicated in Fig. 4. As can be seen in this figure, other islands are located along the muon trajectories. These islands may degrade the quality of tide monitoring and thus this effect will be later discussed.

**Figure 8:**
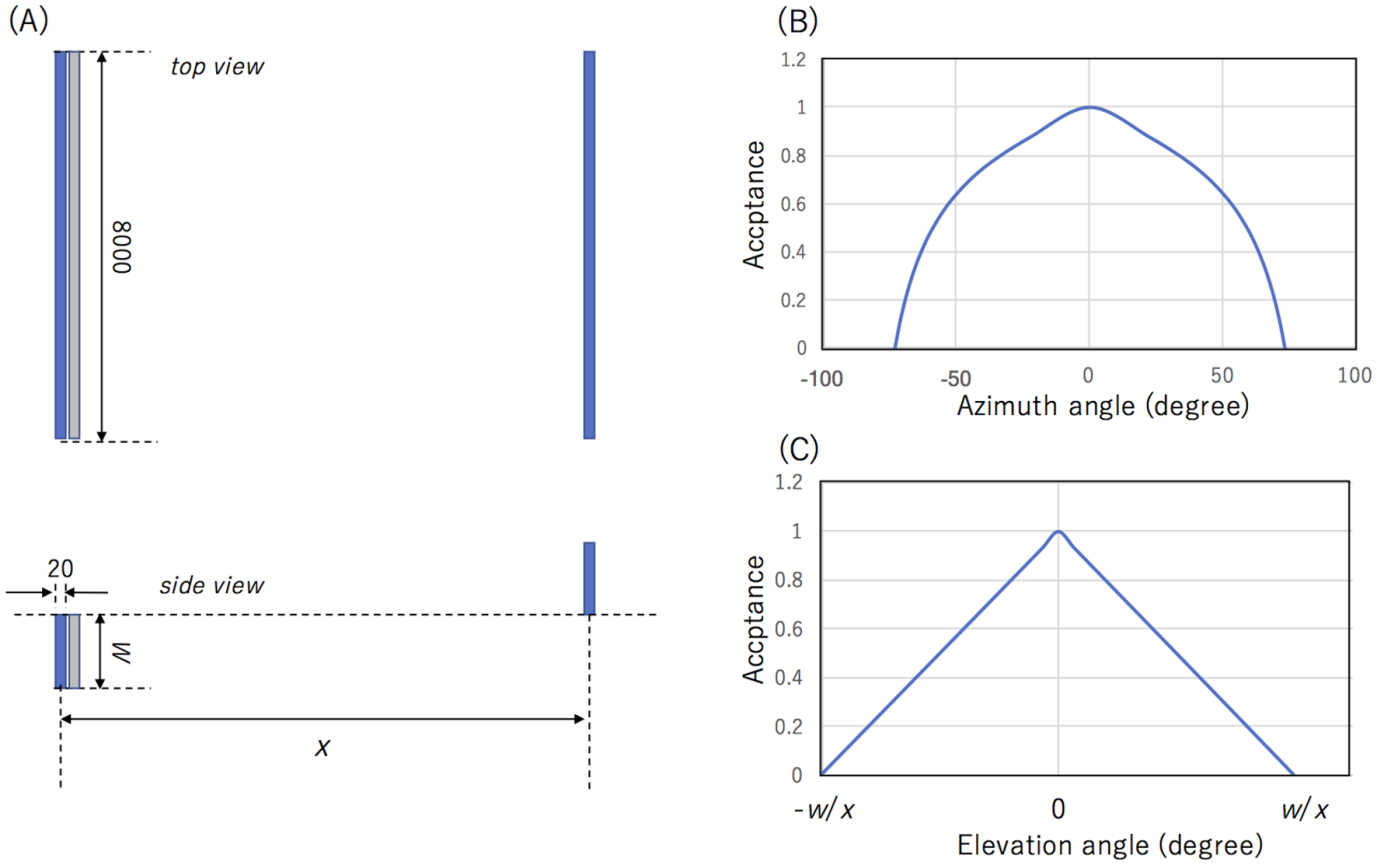
Geometric configuration of HKMSDD-MSM for the tide monitoring network. The top and side views of the setup are respectively shown in the top and bottom panel of (**A**) in units of mm. *x* indicates the distance between the two scintillator strips. The muons arriving from the right to the left side are detected with this configuration. It is expected that the muons arriving from another direction will be sufficiently suppressed (e.g., by a factor of 50 for *w* = 15 cm and *x* = 450 cm) (See Fig. 8). A gray rectangle indicates the 2-cm thick lead plate. The azimuthal (**B**) and elevation angle (**C**) acceptances are also shown for *x* >  > *w*.

Figure 8A shows the proposed HKMSDD-MSM muograph design for the tide monitoring network. HKMSDD-MSM consists of two sets of scintillation detectors that consist of plastic scintillators and photodetectors. The length and the scintillators is 8 m. There are a couple of options for photodetectors: photomultiplier tubes (PMT) or Silicon Photomultipliers (SiPM). In the former case, the PMTs are attached to the scintillators via acrylic light guides. In the latter case, scintillation light is transported to SiPM via wave length shift (WLS) fibers. Since the Eljen's scintillators (EJ-208) and the Kuraray's WLS fibers (Y-11(200)) both have a long attenuation length of 4 m^55^ and 3.5 m^56^, respectively, one photodetector is sufficient for readout of each scintillator strip. Coincidence signals verified to be the same angle between these two detectors are recorded as muon signals. The current HKMSDD-MSM has a wide angular acceptance for the azimuthal angle (Fig. 8B) and a narrow angular acceptance for the elevation angle (Fig. 8C). As shown in Fig. 8A, the scintillator strips are placed so that the HKMSDD-MSM does not receive the muons arriving from the direction opposite to the sea. However, some scattered upward-going muons could generate fake tracks. As shown in Fig. 8C, HKMSDD-MSM does not have an acceptance for the angular region beyond ± *w*/*x*. This design helps to avoid recording muons that didn't pass through seawater, which would eventually degrade the sensitivity to ∆*h*. As was suggested in the reference^15^, a 2-cm thick lead plate is inserted between these detectors to remove background radiation that could emit from the concrete wall of the UUS.

**Figure 9:**
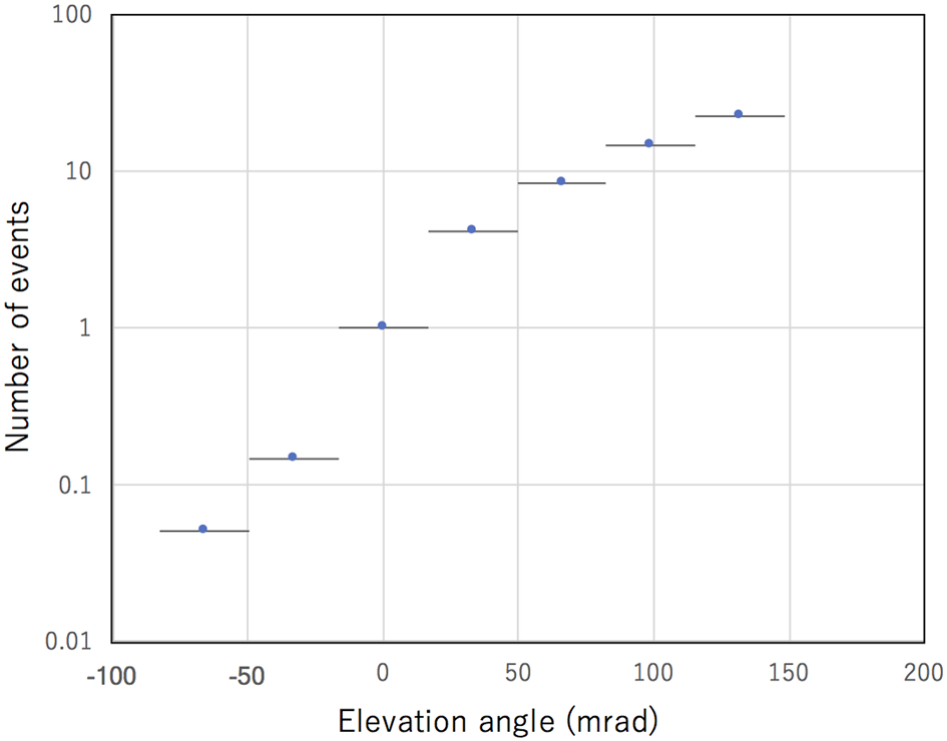
Fraction of the fake tracks generated by the scattered upward going muons as a function of elevation angle. Horizontal bars associated with data points indicate the angular region that occupies 75% of the total acceptance. The statistic errors are within the circles of the data points.

Figure 9 shows the fraction of the fake tracks generated by the scattered upward-going muons as a function of the elevation angles. The number of events were normalized to the value observed at *θ* = 0 ± 16.5 mrad (42 k events in 26 months), where *θ* is elevation angle. The observation conditions to produce this plot are summarized in the Method section. Since these data were taken in the open-sky environment, it is expected that this fraction will be somewhat lower than this in an underground environment. In conclusion, with the currently proposed setup, contamination by the near horizontal backward directed muons will be suppressed to a rate below 1%, and thus this effect will be neglected in the following discussions.

**Figure 10:**
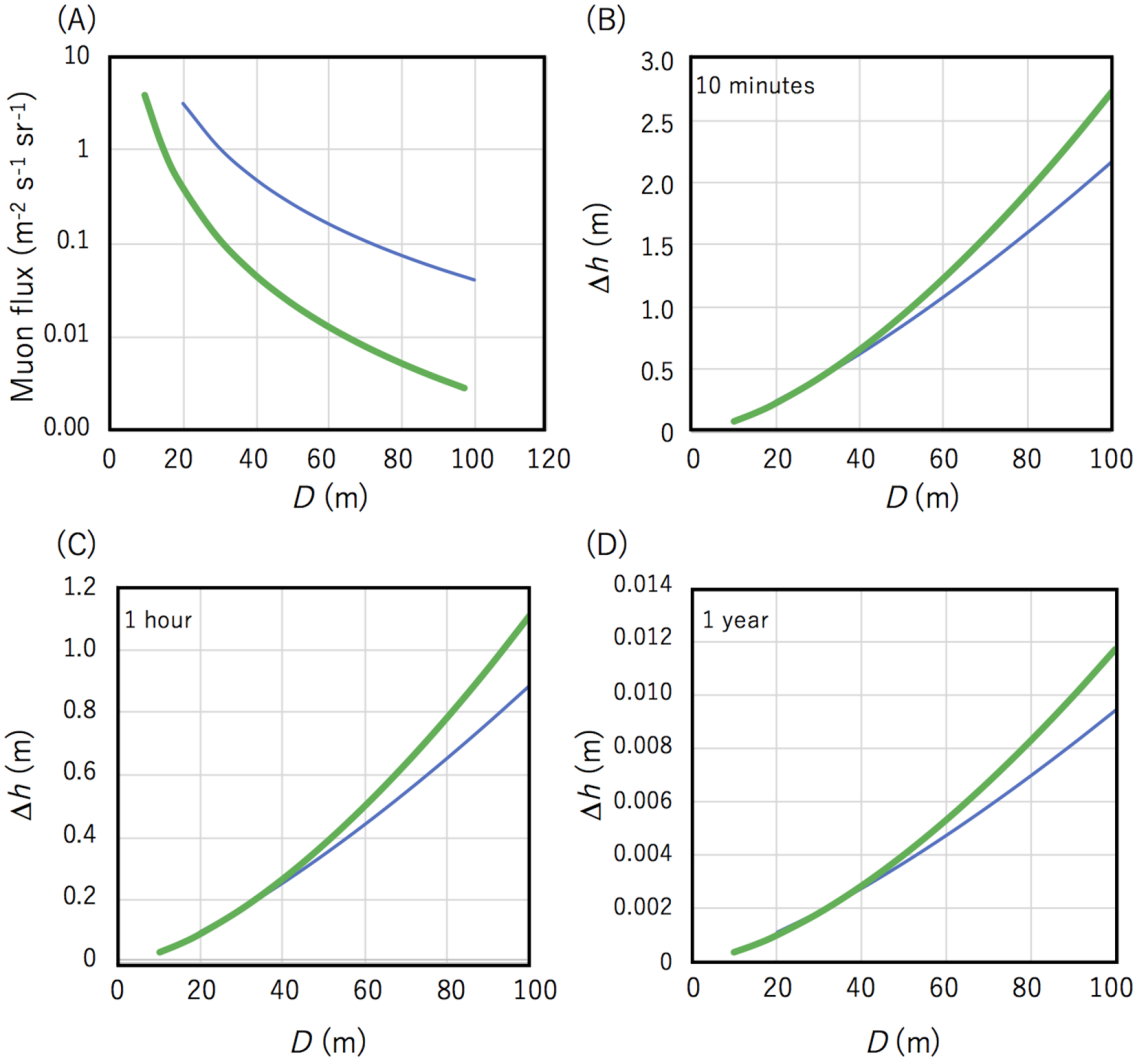
Muon flux (**A**) and detectable tide level variations ∆*h* (**B**,**C**) as a function of the distance between the MSM and the shoreline (*D*) for (*d*-*H*) = 5 m (blue lines), and (*d*-*H*) = 15 m (green lines), where the detectable ∆*h* was defined by the standard deviation of the muon flux. The detectable ∆*h* variations are shown for different time resolutions: 10 min (**B**), 1 h (**C**), and 1 year (**D**).

Figure 10 shows the muon flux and detectable tide level variations ∆*h* as a function of the distance between the MSM and the shoreline (*D*) for different depths (5 m and 15 m) from the mean sea level. Here the detectable ∆*h* was derived from the standard deviation of the number of muons (∆*N*) recorded with the proposed detector configuration (Fig. 8) with the fixed parameters *w* = 15 cm, and *x* = 2.4 m. If the deviation (∆*h*) is sufficiently smaller than (*d*-*H*), the relative penetrating muon flux (∆*N*) which can be expressed as: Δ*N* = *k*Δ*h* + *C* is a linear function of ∆*h*. By utilizing this flux-thickness relationship, the *k* and *C* can be calibrated with the astronomical tide height variations^15^. For the longer value, *D*, since the flux loss by the soil between the muograph and seawater is smaller than flux loss by water, the flux gain by higher elevation and the shorter path length in the seawater compensates for the flux loss due to the soil. Consequently, muographs located at deeper locations would record more muons per unit time; hence would produce better resolutions for determining ∆*h*. As the MSM depth (*d*) increases, the total solid angle required to accept muons at the MSM increases, however, since the ratio ∆*h*/(*d*-*H*) decreases, sensitivity of the ∆*h* detection or time resolution is degraded. These two factors are tradeoffs. The conclusion for dealing with this situation is as follows. For the purpose of real time monitoring (< 1 h), the distance between the MSM and the shoreline (*D*) has to be less than 50 m and 20 m to attain ∆*h* < 50 cm and ∆*h* < 10 cm, respectively. For the purpose monitoring with a longer time scale, if *D* is less than 20 m, sub millimeter accuracy can be obtained per year. From this plot, we also can find that if the MSM depth is shallower, better ∆*h* resolution is achievable for shorter *D*, but better ∆*h* resolution is achievable for longer *D* if the MSM depth is deeper. This is because even the ground soil thickness along the muon path is thicker; hence degrading sensitivity to ∆*h*, for longer *D*, the MSM's acceptance solid angle is larger if the MSM depth is deeper.

**Figure 11:**
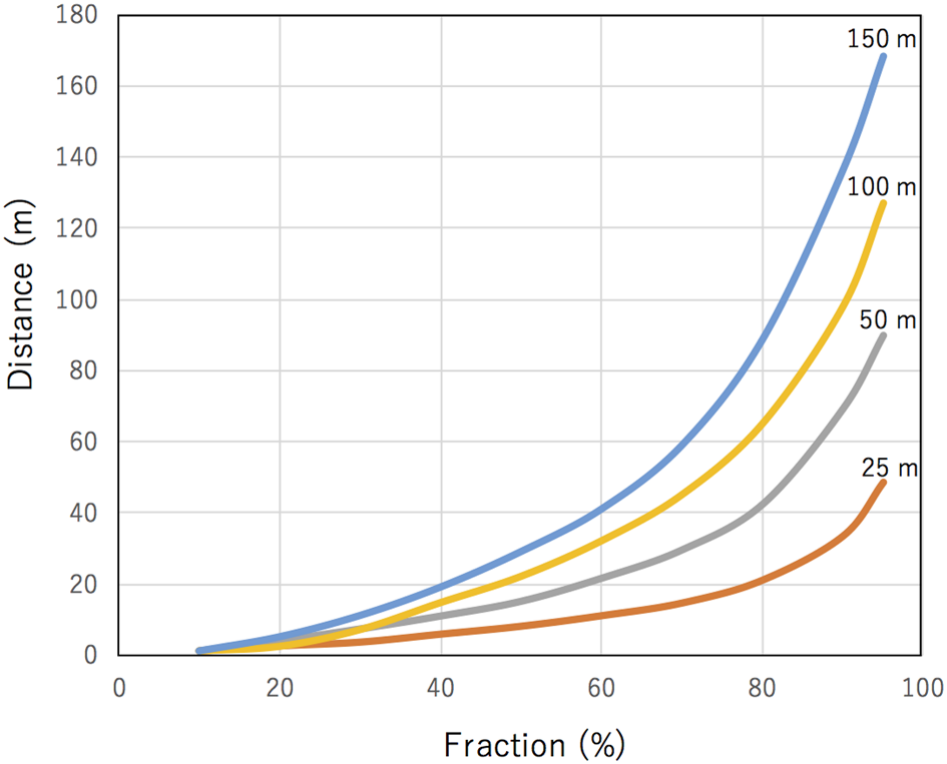
Fraction of the number of muons out of the total number of muons as a function of the distance from the shore line for different distances between the MSM and the shoreline (*D*): 25 m (red line), 50 m (gray line), 100 m (yellow line), and 150 m (blue line).

The current muographic tide monitor measures the tide height averaged over the shoreline to offshore, however, the muon's path length in seawater increases; hence the number of muons decreases as an elevation angle of incoming muons decreases and thus, the observed tide levels are representing those in the near-shore regions. Figure 11 shows the fraction of the number of muons out of the total number of muons (integrated over the entire angular region) as a function of the distance from the shore line for different distances between the MSM and the shoreline (*D*) for (*d*-*H*) = 5 m. As can be seen in this figure, the effect of the islands located further than 200 m from the MSM is negligible. In Cases (A) and (B) (Figs. 6 and 7), by combining the calculation results shown in Fig. 11, combined with the fact that the distance from MSM to the island is more than 180 m, and the depth of the MSM is 5 m from sea level, it is theoretically derived that more than 95% of muons pass through the seawater located between the islands.

## Discussion

Seasonal muon flux variations could affect the sea level measurements. We investigate this possibility and its effect here. The seasonal muon flux variations mainly come from (A) barometric variations^57^ and (B) stratospheric variations^57–59^. Variations in the atmospheric pressure are compensated with the column density of seawater. This inverse barometer effect (IBE) compensation will mostly remove this effect on the sea level measurements. However, the energy loss per unit mass slightly differs between air and water. For example, while the CSDA range is 1.845 × 10^4^ g cm^−2^ for 4 GeV muons in air, it is 1.810 × 10^4^ g cm^−2^ for the muons in water with the same energy^49^. This 2% difference will cause an uncertainty of 5 cm in sea level measurements. The stratospheric seasonal variations generally affect high-energy muons with energies above tens of GeV^57–60^, and may affect the seasonal flux of muons with energies (~ 10 GeV) we are discussing here. This effect will be further studied with longer period measurements at the Tokyo-bay Seafloor HKMSDD.

In urban areas, space on the ground is usually already occupied by buildings and structures, with typically only underground space to utilize as locations for new facilities. Although extra costs are required for ventilation and emergency prevention and response systems, no maintenance of outer walls is needed and, in many cases, underground facilities require less temperature adjustment in comparison to above ground spaces. Moreover, deep underground structures suffer significantly less damage from earthquakes than structures above. Consequently, cities with high population densities tend to develop more UUSs.

The costs required for producing a muographic tide monitor will be less than 6000 US dollars (USD) (3000 USD for a 1-m^2^ plastic scintillator sheet, 2000 USD for 2 PMTs, and 500 USD for a readout electronics unit). Additionally, there would be a power and network connection fee that respectively costs 10–20 dollars/month and 50 dollars/month since the stable power supply with a power generator backup system and the gigabit Ethernet are equipped in the typical UUS in Tokyo. The most costly part is probably the rent of a space, which is an order of 10 k dollars/year for a 100-m^2^ space. However, since MSM itself doesn't require a 100-m^2^ space, it might be possible to share this cost with other tenants. Therefore, the total operational cost for 10 years will be 14 k dollars without rent or at most 114 k dollars with rent. This operational cost is still much lower than the costs (200,000 USD^61^) required for constructing tide gauge stations. For a conventional tide gauge station, the sensors themselves are cheap, but they usually require expensive infrastructures. For example, the conventional tide gauge stations require a land at a harbor, a robust building usually made of reinforced concrete for a long stable operation under harsh environments, reliable electricity and network environments including a backup power generator besides drilling a water well for tide gauging. Also, tide gauge stations cost a lot to operate. For example, it costs more than 30 k dollars just to repair a tide gage station^62^. Alternative techniques, for example, ocean bottom pressure measurements, can use cheap pressure sensors that cost less than 100 dollars^63^. However, in addition to the problems coming from the intrinsic drift of the sensors, the maintenance of underwater sensors is costly. Moreover, for the purpose of monitoring, enormous capital investments need to be made to create underwater network environments for reliable data transfer.

More than 30 nodes of a muographic tide monitor network would be possible within this budget. Moreover, a stainless welded proportional counter (SWPC) currently under development will further reduce the costs for deployment. Since the SWPC structure is simple (consisting of a tungsten wire and a stainless steel tube), the price of one node of the tide monitor network could be reduced to less than 1000 USD in the future. This simple structure also makes it environmentally robust. The HKMSDD-MSM technique is directly applicable to any coastal cities in the world, such as New York, Boston, Miami, San Francisco, Hawaii, Tokyo, Amsterdam, Lisbon, Valencia, Venice, Naples, Marseille, Copenhagan, etc. and it is expected that as cities continue to be more populated, potential muograph locations will also increase.

**Figure 12:**
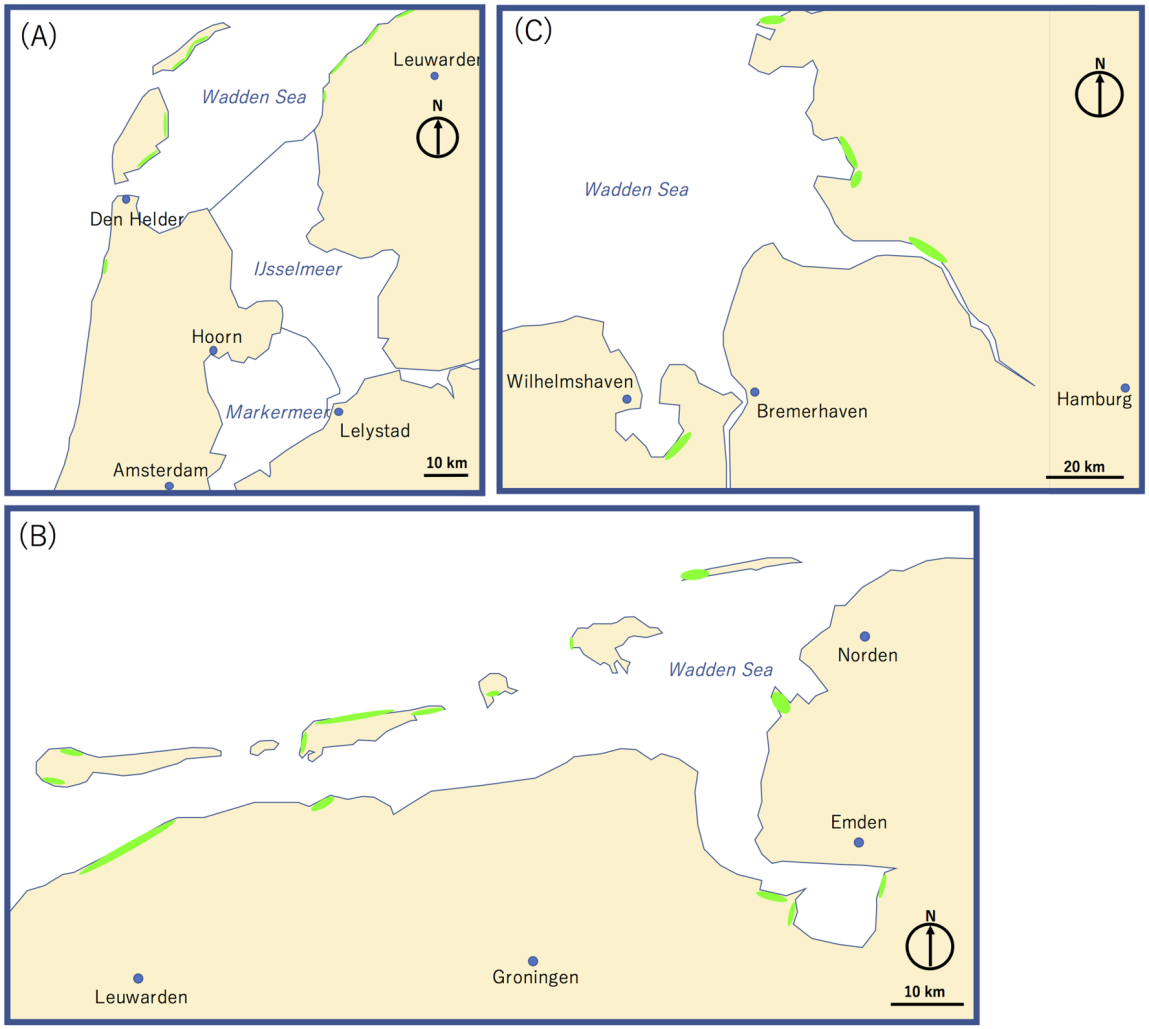
Possible locations of HKMSDD-MSMs in Wadden Sea. The region near Amsterdam (**A**), near the Netherlands-Germany border (**B**), and in the northwest part of Germany (**C**) are shown. The green areas indicate regions that are sufficiently lower than sea level (< − 2 m) along the shore lines, which would be appropriate for the proposed HKMSDD-MSM technique. HKMT drew this map with Microsoft PowerPoint software and holds the copyright.

The currently proposed technique is applicable not only to near-shore UUSs, but also to the land lying below sea level such as many regions of the Netherlands. In such countries, spatiotemporally dense tidal measurements are particularly important for accurate forecasting of storm surges. In the Netherlands, a warning system has been developed and operated by the Dutch storm surge warning service (SVSD) in cooperation with the Royal Netherlands Meteorological Institute (KNMI). The system is based on a numerical hydrodynamic model called the Dutch continental shelf model (DCSM)^49^. Since the 1990s, the accuracy of the system has been improved by incorporating observations of tide gauges. KNMI's automatic production line (APL) has been developed to produce numerical forecasts. However, during the course of this automatic production, the assimilation of only a small number of erroneous observation data will harm the forecast. Figure 12 shows an example of locations in Wadden Sea that would be appropriate for installation of HKMSDD-MSMs. The areas marked along the shore lines are significantly lower than sea level (< − 2 m) and thus, the proposed muographic monitors could be simply placed on the ground floor in any building near shore lines to start tide measurements. By incorporating a low-cost, dense, robust muographic tide monitor network to work in conjunction with the existing system, the quality and accuracy of forecasts would be improved.

Quality of the tidal observations depend on observational density in space and time. With current tidal measurements, high observational density in time is possible but spacial observational density is low. In order to address sub-hourly processes such as meteotsunamis to seasonal and interannual tidal variations, short special scales associated with the high frequency variables have to be covered^21^. The continuity and the smoothness of the spatiotemporal series of total water level, surge and the deviation would be desired and this is achievable with HKMSDD-MSM.

The benefit of a muography-based tide monitoring system in comparison with preexisting systems can be summarized as follows: (A) tide variations can be remotely measured and thus the list of potential locations for safe and stable deployment increases, (B) since sensors do not have to directly touch water (it can be monitored from underground structures and through land/rock obstacles), maintenance costs are more reasonable, and (C) since muographs have no mechanically moving components, the possibility of malfunctions is greatly reduced in comparison to the legacy tide gauge stations, and thus it is easier to realize long and stable operating conditions. On the other hand, the caveat of this muographic technique is (due to the limited muon flux) compromises in either time resolution or sensitivity to tide levels must be made. For this reason, when a smaller detector is chosen for the muographic technique, either time resolution or sensitivity may not be as high as would be possible with conventional tide gauge stations.

In conclusion, due to the high cost and fewer available deployable locations, reliable tide gauge stations can only be constructed in smaller numbers and spatially dispersed. On the other hand, although the temporal resolution and sensitivity of the muographic systems are likely to be inferior to the conventional tide gauge stations, the muographic systems can be more easily deployed and the operational costs are low; thus, a more spatially dense network can be constructed. It is anticipated that by creating a denser network consisting of both the legacy tide gauge stations and MSMs, dramatic improvements to the quality of the surge warning service could be implemented in the near future.

## Method

### Scattered upward-going muon measurements

**Figure 13:**
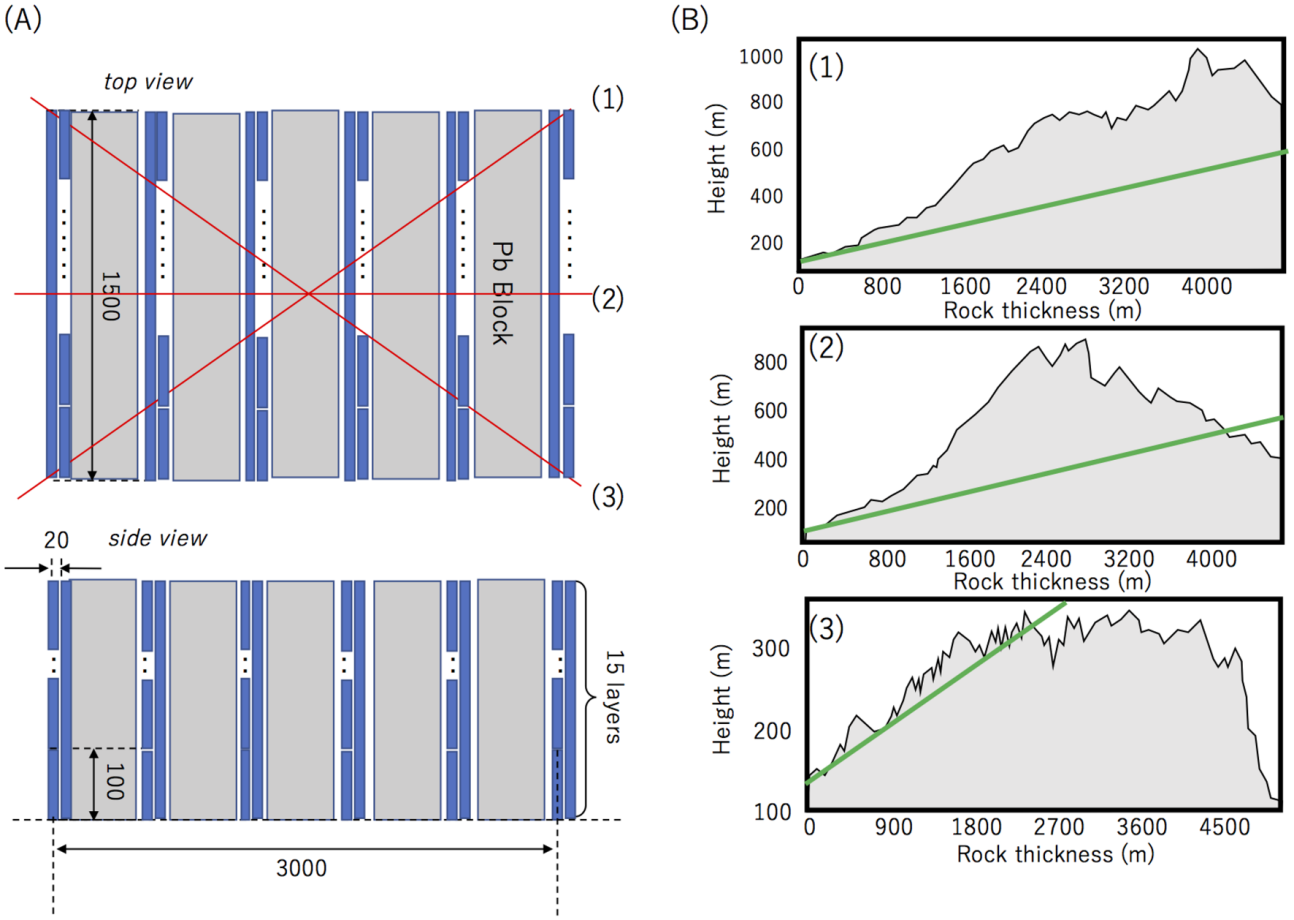
Experimental setup for measuring scattered upward-going muons. The top and side views of the geometrical configuration of the setup is shown in (**A**). The units are in mm. The blue rectangles and gray rectangles respectively indicate MSMs and lead blocks. Red lines indicate the azimuthal viewing angle of the setup. The cross-sectional views of the mountain located in the backward direction are shown in (**B**) for both edges (1 and 3) and the center (2) of the viewing angle. Green lines indicate the trajectories of muons arriving at a 100-mrad elevation angle.

Figure 13 shows the experimental setup for measuring scattered upward-going muons. More detailed explanations can be found elsewhere^37^. The experimental setup consists of 6 MSM layers and 5 lead blocks for radiation shielding. Thickness of each lead block is 100 mm. Each of the MSM layers consists of 15 horizontally and 15 vertically aligned MSMs. Each MSM measures 1500 mm in length, 100 mm in width, and 20 mm in thickness. The distance between the uppermost-stream detector and the lowermost-stream detector was 3000 mm. Therefore, the azimuthal (Φ) and elevation (*Θ*) viewing angle are respectively ± 460 mrad (± 26°) and 460 mrad (26°), the angular resolution was 33 mrad, and only linear trajectories were recorded as muon events. There is a mountain right in front of one side of this setup, but there are no obstacles on the other side of this setup. Here, we define the open-sky direction as the forward direction and the mountain side direction as the backward direction. The rock thicknesses have a tendency to gradually decrease from the direction (1) to the direction (3) in Fig. 13, but they are thicker than 2000 m for the muons arriving the detector at elevation angles less than 100 mrad. Since the flux of the 100-mrad muons after passing through 2,000-m rock is 4.2 × 10^–4^ m^−2^sr^−1^ s^−1^, which is equivalent to 1/2,500 of the open-sky flux of muons arriving at the same elevation angle, the muonic component that directly arrived from the backward direction out of the entire events recorded as backward-directed muons were assumed to be zero for elevation angles less than 100 mrad in this experiment. The plot shown in Fig. 9 was obtained by integrating the number of muons (*N*) over the azimuthal angle range between –Φ and Φ for different elevation angles (*θ*). The geometrical acceptance of the current setup was applied to correct the elevation-angle distribution.
